# Which strategies might improve local primary healthcare in Germany? An explorative study from a local government point of view

**DOI:** 10.1186/s12875-017-0696-z

**Published:** 2017-12-20

**Authors:** Bertolt Kuhn, Kim-Sarah Kleij, Sebastian Liersch, Jost Steinhäuser, Volker Amelung

**Affiliations:** 1 Medical School Hannover, Institute for Epidemiology, Social Medicine and Health Systems Research, OE 5410, Carl-Neuberg-Str. 1, D-30625 Hannover, Germany; 2 University Hospital of Schleswig-Holstein, Campus Lübeck, Institute of Family Medicine, Lübeck, Germany

**Keywords:** Primary healthcare, Communities, Physician shortage, Supplementary care models, Delegation, Mobility, Telemedicine

## Abstract

**Background:**

Facing rising inequities and poorer accessibility of physicians in rural areas, new healthcare delivery structures are being considered to support local healthcare in German communities. To better understand perspectives on and attitudes towards different supplementary models, we examined attitudes among local politicians in the German federal state of Lower Saxony towards the suitability of supplementary care models.

**Methods:**

As part of a cross-sectional study, we surveyed local politicians in Lower Saxony at the local authority and district levels (*n* = 449) by mail questionnaire. We asked for an assessment of four potential supplementary healthcare models at the local level: the use of trained medical assistants, patients’ buses, mobile physicians’ offices, and telemedicine.

**Results:**

The response rate was 71.0% for mayors (*n* = 292) and 81.6% (*n* = 31) for county administrators. In summary, 72.4% of respondents supported the use of trained medical assistants, 48.9% voted for patients’ buses, 22.0% for mobile physicians’ offices, and 13.9% for telemedicine. Except for telemedicine, the politicians’ approval of the supplementary models in rural areas was higher than in urban areas. The assessment regarding the suitability of each model was not significantly connected with indicators of a positively or negatively assessed local healthcare situation. The analyses showed that the use of trained medical assistants was associated with the positive effects of division of labor and potential to relieve physicians. In contrast, there was skepticism about technical support via telemedicine, mostly due to concerns about its unsuitability for elderly people and the potential lower quality of healthcare delivery.

**Conclusion:**

Local politicians widely accept the use of trained medical assistants, whereas the applicability of technical solutions such as telemedicine is perceived with skepticism. Therefore, the knowledge gap between evidence for and prejudices against telemedicine needs to be addressed more effectively. Reasons for the assessments of the presented models are more likely traceable to personal views than to assessments of the actual estimated local primary care situation.

**Electronic supplementary material:**

The online version of this article (10.1186/s12875-017-0696-z) contains supplementary material, which is available to authorized users.

## Background

In Germany, ambulatory care physicians are traditionally self-employed. However, their distribution is regulated and allocated by the association of statutory health insurance physicians, which is responsible for maintaining a sufficient and high-quality supply of physicians. By federal law, there are different regulations and planning areas for general practitioners and other medical specialists. The distribution aims at achieving a predefined ratio between physicians and the number of people who live in the specific planning area. There is, for example, one full-time working general practitioner (GP) for every 1671 people in a particular planning area [1]. Regions are defined as oversupplied when they have a number of physicians that exceeds the respective reference ratio by more than +10%. In such cases, further settlements of physicians in these areas are blocked. In contrast, physicians are incentivized to settle in regions that are undersupplied. Undersupply is defined as a physician-population ratio below the predefined ratio of −25% for GPs and −50% for specialists [2]. The so-called needs-based planning method was created in the 1990s in response to the increasing number of physicians in Germany, at a time when the buzzword “physician glut” was circulated [3]. Therefore, the spatial planning of physicians mainly aims at equalizing their distribution, with restrictions used to prevent oversupply. However, the planning instruments have turned out to be less suitable for placing new physicians in undersupplied areas. Because physicians can find attractive jobs in cities, in hospitals, or in the non-curative sector, the restrictions on settlements are not forcing doctors to work in rural areas [4].

The distribution of physicians in Germany is not controlled by the type of price competition that would lead to lower incomes for doctors in areas with a high density of providers. Physicians are remunerated according to an official fee scale and receive fixed prices for the provision of individual services. There are separate medical fee scales for patients within the statutory health insurance scheme and those with private health insurance. Fees for privately insured patients are higher; thus, doctors with a higher percentage of private patients usually earn more money [5]. This leads to a politically unintended incentive for physicians to prefer areas with a high share of privately insured people, regardless of the need structure.

At the moment, the supply of healthcare in Germany is still considered good. For a long time, no specific requirements were considered for rural healthcare due to the relatively similar conditions across regions. Compared to other countries, Germany’s physician supply and infrastructure are considered to be quite well established. Specialized doctors seem to be acceptably distributed, despite the aforementioned problems and negative developments [6].

Nevertheless, nationwide availability of physician care will be threatened in the future by distinct misalignments between urban and rural areas. Particularly, the following three factors are strengthening the discrepancies between urban and rural areas in Germany:

1. Societal transformation of the doctor’s profession. Among physicians, there is a growing desire for flexibility, good working conditions and shorter working hours that will potentially lead to a poorer operational output on the part of an average physician in relation to the population. Instead of being self-employed as rural physician in their own offices, young doctors are increasingly choosing to work for hospitals or as employees in medical service centers in the ambulant sector [7]. These opportunities are mainly located in urban regions.
2. Poorer access to physicians’ facilities in rural areas. In rural areas, the travel times for a patient to reach a physician tend to be longer [8]. Often, there is lack of appropriate public transportation. Access to healthcare services is often lower than in urban areas, which leads to challenges for people who are dependent on public transport, especially if they are immobile due to health problems or disabilities [9]. As local transport in rural regions with shrinking populations is limited for economic reasons, problems with access to medical care are increasing in certain regions.
3. Growing number of older people in rural areas. A rising number of older people correlates with an increasing number of people with mobility issues and health problems who need adequate access to medical care [6].

Despite the regulations on physicians’ settlements, there is already a considerably lower quantitative physician-population ratio in rural areas [10]. A psychotherapist in the rural district of *Holzminden,* for example, may potentially provide care for 4.4 times as many people as a psychotherapist in the city of *Göttingen* [11]. Regardless of the prevailing opinion that the situation is still acceptable in most areas, the long-term trends are worrying and considerable. Problem-solving approaches require a long lead time before they can have an effect [7]. If too much time elapses without action, there is fear of a downward spiral that will lead to an exodus of rural physicians [10].

Differences in healthcare provision between rural and urban areas are common in Europe, although the extent of the disparity varies from country to country [7, 12]. There are three main strategies with which policy makers can address current and future challenges in response to geographic imbalances in physician supply:

1. Target future physicians: Increase the pool of future physicians who are interested in practicing in underserved regions (e.g., target aspects of medical education).
2. Target current physicians: Maximize the share of current physicians who are willing to practice in underserved areas (e.g., increase the attractiveness of resettling in specific regions through higher wages and subsidies).
3. Do with less: Change existing supply structures to enable more efficient care services so that fewer doctors are required for the delivery of medical services (e.g., implement new service delivery models) [7].

There are rising concerns about finding replacements for retiring physicians and about attracting young physicians to rural areas. Because undergraduate medical studies are usually completed at universities in German cities, students often have little or no contact or practical experience with rural medical care during their education. In addition, training in family medicine is rather unattractive for medical graduates in Germany compared to graduates in other European countries [13, 14]. Particularly for populations in small communities in rural areas, accessibility problems will probably increase due to the reduced number of physicians’ offices [15]. In Germany, efforts targeting future and current physicians are typically organized at the national or federal state level. The local level provides a testing ground for new supplementary health care services to tackle specific local needs. If the care supply is going to be more diverse and the situation worse in certain areas, then there is pressure to take specific actions. As a result, new heterogeneous approaches to health service delivery can be tested at the local level in combination with traditional supply models [16]. The main problems seem to be overloaded doctors and access problems for patients. Promising short-term approaches on the local level focus on relieving pressures on physicians by obtaining help from other professional groups, as well as using mobility and telemedicine models to bring patients and doctors together [10].

German communities and municipalities have no direct responsibilities for ensuring that local healthcare is provided by physicians. However, communities and municipalities are affected if the local population faces problems because of insufficient healthcare delivery, long travel times, or other accessibility factors resulting from a local physician shortage [17]. Due to these developments, communities and municipalities are playing a more important role in ensuring local healthcare supplies and satisfying the needs of the population [18]. The municipal governments recognize the specific local conditions and challenges related to healthcare utilization. They are able to support the settlement and distribution of physicians by using different approaches and measures [7]. Models, such as delegation models, new forms of mobility, and telemedicine, are thought to be suitable and are promoted at the federal level [10]. We wanted to know whether these assessments are also reflected at the local level. German municipalities typically make policy decisions by coordinating executive initiatives with the majority of the local council. This process is strongly influenced by the constraint of fiscal consolidation and by the need to avoid resistance from citizen protests [19]. Policy makers are further influenced in decision making by forces such as the opinions of a dominant epistemic social environment or narrative [20]. These aspects, therefore, also contribute to local politicians’ assessments of potentially relevant supplement concepts in primary care.

In conclusion, our study aims to investigate local politicians’ perspectives on the following questions:

1. What expectations are associated with supplementary care delivery models?
2. Which models are accepted as potentially suitable to ensure healthcare delivery in communities?
3. Are supplementary models more likely to be seen as suitable if local healthcare is assessed as poor?

## Methods

### Supplementary care models

**Fig. 1:**
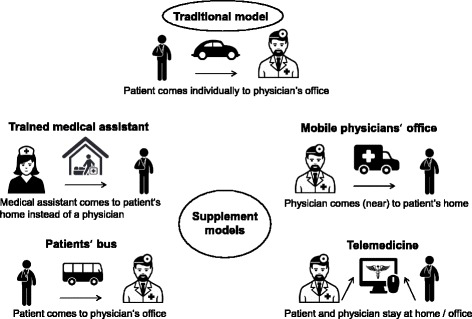
Basic characteristics of the supplementary models. Icon sources: *Designed by Freepik and distributed by* *Flaticon.com* *& Shutterstock*

We surveyed assessments of four supplementary care models considered to be innovative in Germany. The selection of supplementary care concepts was based on models that have been identified as potentially favorable and on concepts for improving outpatient medical care that have been partially implemented by the German council of experts on the assessment of health care developments [10] and current regulatory initiatives. This choice of models was also oriented towards a simultaneously conducted population survey with a similar thematic focus [21]. The following supplementary models complement the existing healthcare supply and do not replace the available infrastructure. They can potentially increase the accessibility of health services and lead to a decrease in the need for home visits by physicians. A visual summary of the supplementary models is given in Fig. 1.

### Trained medical assistant

Models that include the delegation of medical services to qualified non-medical health professionals have been disseminated internationally [6]. The core idea is to allow non-medical healthcare professionals to perform tasks that are traditionally performed by physicians [22]. The desired result is to enable doctors to focus on their original medical activities, which leads to a reduced workforce requirement for physicians [23, 24].

The outpatient healthcare system in Germany is generally centered on physicians, who have a relatively large range of activities. Other healthcare professionals rarely have academic qualifications [1]. From an international point of view, there is currently no standardized way to qualify as a nurse practitioner in Germany. However, German physicians delegate certain activities to other professionals, such as qualified nurses or medical assistants with further education [25]. Since the mid-2000s, delegation models have been implemented in which qualified nurses or medical assistants autonomously perform home visits and some medical activities. This has been done in response to looming supply shortages due to overloaded physicians and due to an increasing need for treatment because of demographic changes and the rising prevalence of chronic diseases [26]. In the remainder of this paper, these additional qualified healthcare staff members are called “trained medical assistants” (TMA).

### Patients’ bus

A growing problem that impedes access to medical care is the limited public transport in rural areas. Existing public transport services are often aligned with school transport and operate according to school hours and school holidays. Partly, there is a need for alternative modes of transportation to meet the growing need for patient transport to places that provide medical services [27]. Especially in rural areas, people without their own cars or driver’s licenses are dependent on appropriate public connections to medical care. Models, which can be summarized under the term “patients’ buses”, have in common that they provide public transportation from the patient’s residence to physicians’ offices. A patients’ bus can be organized as a demand-based on-call bus or as a scheduled service bus with voluntary or vocational drivers [10].

In the German federal state of Brandenburg, the association of statutory health insurance physicians, in cooperation with the municipalities, developed a project to ensure access to physicians via public transport in one county. Once a week, a minibus brought patients directly from eight districts to different physicians’ offices in the center of the neighboring larger city. The traffic connection was arranged according to the doctors’ office hours [28].

### Mobile physicians’ office

Mobile utilities offer a range of physicians’ services as a community-based medical supply base. They aim at avoiding regional undersupply; thus, they usually consider specific vulnerable populations (e.g., socially disadvantaged individuals, migrants or older people) [29].

Generally, in Germany, mobile utilities are rarely used to deliver primary healthcare services. In 2013, there was a pilot project in which a utility van was converted into a GP office. Different physicians approached six small communities that lacked an existing GP office [30]. A follow-up project with the same van aims to ensure low-threshold primary care for refugees in the federal state of Schleswig-Holstein [31].

### Telemedicine

Telemedical concepts include the provision of healthcare services from a remote location using electronic information and communication technologies [32]. Telemedicine focuses on medical situations in which the personal appearance of the patient in a physician’s office is not necessary, e.g., to discuss indications or to obtain a second opinion based on medical findings. In the context of this study, telemedicine is understood in terms of telecare, which means the provision of medical care by a doctor who is at a distance, allowing patients to be treated in their own homes.

Particularly for rural regions, telemedicine is assessed as potentially suitable as a supplementary service model. In these areas, less infrastructure is maintained, and long distances can be bridged spatially with electronic devices [7, 33]. At present, there are approximately 200 pilot projects using telemedicine approaches in the existing German care delivery structures. However, very few tested telemedicine concepts have been transferred to standard care [33].

### Questionnaire and survey

We developed an experimental questionnaire (Additional file 1 **,** Additional file 2). Pretests were performed to test the questionnaire’s suitability. Therefore, interviews were conducted with representatives of local government associations in the federal state of Lower Saxony (*n* = 2) and with mayors (*n* = 5) in neighboring federal states.

In September 2015, a postal survey was conducted. In accordance with the aims of this study, community politicians were questioned regarding three aspects of municipal healthcare:

1. Current local physician supply;
2. Importance of outpatient healthcare for municipalities and assessment of supporting measures;
3. Attitudes towards innovative care models to support outpatient healthcare (trained medical assistants, patients’ buses, mobile physicians’ offices, and telemedicine).

**Fig. 2:**
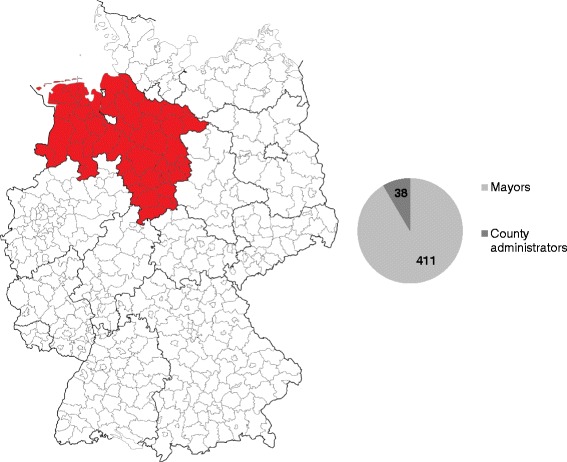
Study population in Lower Saxony. Map modified from [49]

The study sample consisted of all professional mayors (*n* = 411) and county administrators (*n* = 38) in the federal state of Lower Saxony in North-Western Germany (Fig. 2). Lower Saxony is the second largest German state by land area and, with a population of approximately 8 million people, the fourth largest state by number of inhabitants in Germany [34]. The people surveyed were the directly elected policy leaders at the community and county municipal levels.

Data gathered in the study were collected anonymously. We asked about several socio-demographic characteristics of the participating mayors, including age and sex. Due to reasons of anonymization, we did not collect these data from the smaller group of county leaders.

Different classifications and definitions of rural areas are used in the literature [17]. We examined the subjective assessments of the mayors regarding whether their community was located in an urban or densely populated area or a rural area. We were not able to double-check these assessments due to the anonymous character of the survey. In the following, the definition of rural and urban only refers to this subjective classification.

### Analysis

The questions were mostly answered on Likert scales that had either four or five options. In addition, the questionnaire included open and semi-open questions. The answers were partly summarized dichotomously (e.g., approval, no approval) for evaluation.

Depending on the level of measurement of the specific variables, we used the Pearson Chi-Square test, the Mann-Whitney U test, Spearman’s rank correlation test, and binary logistic regression. A difference at the level of *p* < 0.05 indicates significant results in all performed tests. Shares are given in valid percent. We used SPSS version 23 for the statistical analyses.

We asked the respondents to briefly provide the reasoning behind their given assessments of each supplementary model’s suitability. For the categorization, the answers were assigned into three groups according to the related suitability assessment: the 1st group was “positive assessment” (suitable & rather suitable); the 2nd group was “neutral or undecided assessment” (partly / partly); and the 3rd group was “negative assessment” (not suitable & rather not suitable). Next, free text answers were assigned to content categories using MAXQDA version 11 and Microsoft Excel for a basic qualitative evaluation. These categories were defined inductively and revised in the course of the assessment process [34]. Two people performed the categorization parallel to and separately from each other. After discussion, we reached a consensus with a final common category scheme.

## Results

The response rate was 70.9% for mayors (*n* = 292) and 81.6% (*n* = 31) for county administrators. The sample was representative of Lower-Saxony with regard to the sex of the mayors and the size of municipality by population classes. A total of 81.3% (*n* = 230) of the participating mayors stated that their community was located in a rural area, which is significantly more than the official spatial distribution in Lower-Saxony, where 57.7% of communities are rural.^1^

**Fig. 3:**
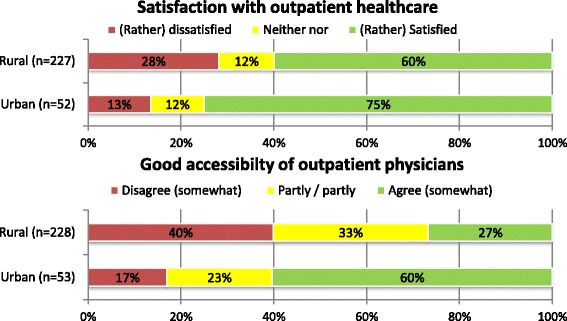
Satisfaction & assessment of good accessibility regarding outpatient healthcare in urban and rural areas

Predominantly, the respondents were satisfied with the local outpatient healthcare, and the situation in urban areas was evaluated slightly more positively than that in rural areas (*p* < 0.001) (Fig. 3). The assessment of satisfaction correlated weakly but significantly with the size of the community (Spearman-Rho = 0.17, *p* = 0.007): Mayors of communities with larger populations tended to be more satisfied with the ambulant physician’s care.

For each municipality, the inhabitants’ ability to access physicians by public transport was assessed differently depending on the spatial location of the respondents: In urban areas, the majority of participants agreed that accessibility was good, whereas in rural areas, most of respondents disagreed (p < 0,001) (Fig. 3).

**Fig. 4:**
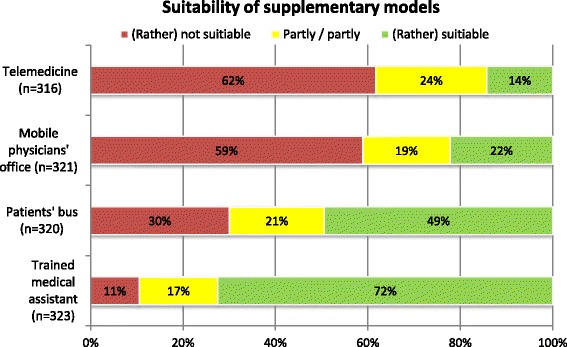
Summarized assessment of supplementary models in health care

Asked about possible approaches to improving healthcare delivery, the local politicians evaluated the suitability of the four supplementary models for their respective settings quite differently. In summary, 72.4% of the respondents supported the use of a TMA; 49.4% voted for patients’ buses, 22.1% for mobile physicians’ offices, and 14.2% for telemedicine (Fig. 4).

The comparison of the mean assessment showed that the presented supplementary models were seen as more suitable in rural communities compared to urban ones, except for telemedicine (TMA: *p* = 0.018; patients’ bus: *p* = 0.001; mobile physicians’ office: p = 0.001; telemedicine: *p* = 0.799).

We calculated a binary logistic regression model for each of the supplementary models to investigate whether communities with inferior infrastructure and poorer assessments of the actual care situation are more open to the supplementary models (Additional file 3). We used the dichotomized assessments of the suitability of each supplementary model as dependent variables and simultaneously added several covariates and socio-demographic variables to the model. The covariates served as indicators of a more poorly assessed outpatient healthcare situation. As a result, the regression model indicates that the evaluation of the suitability of each supplementary model is not significantly correlated with most of the covariates. The levels of the coefficients of determination of the model range from 0.040 for telemedicine to 0.068 for a TMA (Nagelkerke’s R-Squared). Thus, the model fit can be observed as having very low explanatory power for the suitability assessment of the supplementary models.

According to the free text responses, the use of TMAs was often associated with advantages of the division of labor, which could potentially lead to more consultation time for patients and improved availability of home visits. For some respondents, there was also the expectation that a skill transfer from physicians to TMAs could improve the working conditions and satisfaction of both professional groups. Negative arguments were that a TMA would not be a substitute for a physician, and some responses expressed a lack of acceptance and trust in a TMA.

The feelings about patients’ buses were mixed. The need for public transportation to physicians’ practices was noted widely. Some argued that there was no need for buses because of existing public transport. The additional effort required might be too great and not suitable for the respective community structure. On the other hand, some communities reported similar alternative public transport models based on civic engagement, and these were assessed positively.

The need for mobile physicians’ offices was predominantly denied. A majority believed that patients’ acceptance might be low and that the necessary financial effort would be too high. Furthermore, the driving time required of the physicians, at the expense of treatment time, was remarked on negatively. However, a few respondents thought the model could be suitable in rural areas or specific spatial community situations as a limited workaround.

Telemedicine models were frequently associated with an impersonal treatment model, and there were broad reservations about this approach. Often, it was argued that this model would not be suitable for elderly patients who would be averse to technically supported non-physical contact with doctors. In addition, there were reservations about the availability of the technology and the quality of care. Positive arguments were the possible advantages for certain patient groups, particularly for patients with specific groups of diseases. A few participants argued that telemedicine could represent a model for future generations.

A summary of the main arguments about and associations with the supplementary models is presented in Table 1.

**Table 1:** Summary of categorized arguments about and associations with the supplementary models

Trained medical assistant	Positive	Work-relief for physicians (*n* = 54)	Improve treatment quality and care (*n* = 17)	Support for the provision of home visits (*n* = 12)	Appropriate tasks for delegation (n = 12)	Positive experiences with similar concepts (*n* = 11)	Remedy for physician shortage (*n* = 7)
	Negative	Lack of acceptance and trust (*n* = 10)	Not an adequate substitute for a doctor (n = 7)		No general need (n = 6)	Concerns about treatment quality (n = 5)	
Patients‘bus	Positive	Good addition to existing public transport (*n* = 25)	Similar public transport systems available or planned (*n* = 21)		Suitable to ensure mobility and accessibility (*n* = 19)	Suitable supplement for rural and widespread areas (*n* = 14)	
	Negative	Sufficient mobility offers available (n = 25)	High effort and costs (*n* = 16)	Not suitable for the specific community and settlement structure (n = 12)	Low demand and utilization projected (n = 6)	Not necessary due to good accessibility or short distances to medical practices (*n* = 6)	
Mobile physicians‘office	Positive	Suitable supplement for rural and widespread areas (n = 16)			Sufficient to secure the supply of medical treatment (n = 7)		
	Negative	Lack of acceptance and trust (*n* = 39)	Not necessary due to good accessibility or short distances to medical practices (*n* = 20	Not suitable for the specific community and settlement structure (n = 20)	No general need (*n* = 15)	Concerns about treatment quality (*n* = 9)	Inefficient use of doctors due to travel expenses (*n* = 9)
Telemedicine	Positive	Suitable for specific group of persons (n = 10)		Forward-looking model with potential (n = 7)		Suitable for specific indications (n = 6)	
	Negative	Not suitable for older people (*n* = 57)	Impersonal type of treatment (*n* = 33)	Poor availability of necessary technology (n = 29)	Concerns about treatment quality (n = 16)	Lack of acceptance and trust (n = 14)	

## Discussion

In this study, the participants were mostly confident that a TMA would be able to deliver high-quality treatment as part of their responsibilities. The potential relief of doctors through the division or delegation of labor was estimated broadly. The positive assessment of a supporting model with a greater role for TMAs coexists with the rising trend of implementing such models in the German ambulatory healthcare system to support non-medical professions in primary care [35]. In recent years, there have been several projects to expand the scope of activities and responsibilities of non-medical personnel in Germany [26]. The expansion and acceptance among patients of TMAs and nurse practitioners can also be observed in the USA [36] and other countries with highly developed primary care infrastructures [25].

The weak acceptance of technical solutions by the respondents is notable. It was often said that these models would not be suitable for older people. Participants’ broad rejection of telemedicine as a complementary care model contrasts with international developments, which promote a greater dissemination of technical solutions and anticipate several advantages, especially for older patients [37, 38]. The expressed skepticism of the survey participants in our study was also observed in patients’ surveys [39]. In contrast, consultations between physicians and their patients via telephone or internet devices in order to provide remote healthcare advice are increasingly in demand among patients in comparable European countries such as France, Switzerland and the United Kingdom [40]. Additionally, there is growing evidence in favor of telemedicine regarding clinical outcomes and improved quality of services [41, 42]. A US-American study showed a significant correlation between rising age and an unfavorable attitude towards the use of telemedicine [43]. This corresponds with the often-cited expectations of the municipal leaders that telemedicine would not be suitable for older populations. Rural areas may lack high-speed internet connections, which is an important requirement for the diffusion of telemedicine [7]. Thus, the promotion of fast and reliable communication infrastructure in Germany should be recognized as an important precondition for telemedicine. The results might also be interpreted as reflecting a fear among municipal politicians that technical instruments will have too much decision-making power at the expense of an accurate personal verification of patients’ needs.

Community leaders had different opinions regarding the implementation of transport solutions such as patients’ buses intended to transport patients to physicians’ offices. It seems to be common sense that physicians’ offices are important destinations for public connections. Generally, transport options other than patients’ buses seem to be favored. One important aspect of the suitability of patients’ buses could be the financial requirements and related municipal involvement.

Mobile physicians’ offices were predominantly seen as not suitable; at best, they were seen as the option of last resort for undersupplied communities. There are examples of mobile utilities in other supply areas that have been partly accepted as a way to overcome local supply gaps: e.g., mobile libraries or mobile citizen centers [44]. In medical treatment, there is prevailing skepticism. Often, no need was observed for mobile care solutions, which can perhaps be attributed to a sufficiently dense network of resident doctors in Lower Saxony. According to a study of German medical students, future physicians are potentially quite willing to work in a mobile physician’s office [45].

As mentioned above, the Lower Saxony municipal survey collected assessments of supplementary care models, which were also collected in a population survey in Lower Saxony. In both surveys, medical delegation models were relatively frequently accepted as a care option, while a telemedicine treatment contact was mostly not accepted. In the general population, greater acceptance of a mobile doctor’s practice compared to a patient’s bus was observed [21].

Depending on the need and respective care situation, supplementary care models could be applied individually or in combination. There is not necessarily a trade-off between a TMA and telemedicine. Rather, both, in combination, could support effective healthcare delivery in undersupplied areas [46]. The described models tackle problems of local accessibility in healthcare. However, they do not ensure a sufficient number of doctors. The supplementary models could only help to improve the need-based utilization of physicians if the available physicians are not yet fully occupied. This also applies to delegation models with a TMA, whereby the supervision and cooperation of a physician is necessary, which limits the relief provided to the physician [47].

The binary logistic regression showed little evidence that the supplementary models are more favored if the local physicians’ care situation was assessed negatively, with the chosen indicators as covariates. Thus, our model is not appropriate to explain the differences between the overall assessments. This suggests that more than situational and location factors – and not the queried personal opinions and views of the participants – are causing differences in the suitability assessment.

The bivariate analysis of the relation between the spatial classification of the community and the assessed suitability of the supplementary models showed significant differences for the models, with the exception of telemedicine. In multivariate analysis, the spatial classification only had a significant influence on the assessment of the patients’ bus model.

### Strength and limitations

The study contributes evidence from the local politicians’ point of view to current discussions about supplementary models in primary healthcare delivery. This study offers another important perspective – in addition to the patients’ perspective – on different innovative approaches. To our knowledge, this survey is the first to focus on the target group of German municipalities and their attitudes towards supplementary models of primary care delivery.

The study’s strengths also lie in the high response of more than 70%. Thus, the findings can be considered representative for Lower Saxony. Lower Saxony can be considered representative of the overall German settlement and healthcare supply structure due to its diverse settlement structure, which includes areas that are very rural as well as large centers. The results can therefore be regarded as nationally and internationally transferable.

There are also some limitations that should be considered.

The developed questionnaire was experimental. The supplementary care models were explained in the questionnaire with brief descriptions and illustrating pictures. It remains unclear what additional knowledge the local politicians had about each model and how this potential knowledge influenced their responses. Although the questionnaire was thoroughly tested previously, the descriptions may have been misunderstood by the respondents in some cases.

The opinion of the surveyed respondents towards the supplementary models was assessed on a fundamental level to capture their first impressions of an innovative approach to outpatient care. The analysis shows the expressed basic expectations and reservations of the local authorities. Important aspects, such as financial questions, potential municipal participation, and willingness to pay, were not included in the questionnaire. However, financial aspects certainly matter for a local implementation of supplement concepts and have been partially included in the answers regarding local suitability. In total, the study’s findings are not appropriate for use as a political basis of decision-making. For this reason, the results should not be used as arguments for or against the suitability of a model but rather as indications of the personal points of view of local authorities, which contribute to the discussion at the federal level.

Our investigation was based on a cross-sectional study, which has its limitations, such as the selection bias of the respondents. Therefore, the results cannot necessarily be generalized to future sampling.

## Conclusions

This study provides information on how local politicians generally contrast different supplement models in primary care and with what arguments and associations the different models are linked. Although these aspects are not usable as broader policy advice, the analysis provides an inventory of existing assumptions and prejudices at the local level, which should be taken into account when implementing supplementary care models.

Although there are significant differences between urban and rural areas, overall, the mayors and county leaders in Lower Saxony are more satisfied than dissatisfied with the local medical care situation.

The survey respondents estimated the practicality of the respective supplementary models very differently. From the politicians’ point of view, increased service from non-medical staff such as TMAs is well accepted, and not only in response to a physician shortage. Extended public mobility models that focus on primary care seem to be an important issue for many of those surveyed. For some respondents, patients’ buses may be considered a possible useful option, whereas the suitability of mobile physicians’ offices is predominantly rejected. The presented telemedicine model has widespread negative associations. If telemedicine approaches are to be implemented, the barriers shown here at the local level should be taken into account.

Currently, for the majority of the population, doctors’ offices are still relatively easy to access, for both the urban and rural population. There are usually more barriers to structural changes than to keeping the status quo. The question is whether the assessment of supplementary models will change when the situation worsens in certain areas, as forecasts and social trends predict. Perhaps, the need for physicians must become more severe before the association of statutory health insurance physicians and municipalities will leave the familiar situation behind and try unconventional approaches such as alternative models of access to healthcare.

## Additional files

Additional file 1: Questionnaire mayors. The translated questionnaire for the survey of mayors in Lower Saxony. (PDF 493 kb)

Additional file 2: Questionnaire county administrators. The translated questionnaire for the survey of county administrators in Lower Saxony. (PDF 484 kb)

Additional file 3: Binary logistic regression model of the suitability of the supplement models. The regression model investigates possible connections regarding the suitability of supplement models with with indicators of a positively or negatively assessed local healthcare situation. (DOCX 15 kb)

## Abbreviations

GP: General practitioner
TMA: Trained medical assistants

## Footnotes

1 Spatial classification in accordance with the criterion of population density (<150 inhabitants / square kilometer) [48]
