# Supplementary material for: Which strategies might improve local primary healthcare in Germany? An explorative study from a local government point of view
Source: BMC Fam Pract. 2017 Dec 20;18:105. doi: 10.1186/s12875-017-0696-z (PMC5738820; doi:10.1186/s12875-017-0696-z)
Supplement: Supplementary file 2 — Questionnaire county administrators. The translated questionnaire for the survey of county administrators in Lower Saxony. (PDF 484 kb) [file 12875_2017_696_MOESM2_ESM.pdf]

Institut für Epidemiologie, Sozialmedizin und Gesundheitssystemforschung

Medizinische Hochschule Hannover

Institute for Epidemiology, Social Medicine and Health Systems Research
MHH – Hannover Medical School

Survey of County Administrators in Lower Saxony on the Municipal Impact of Outpatient Physician Supply

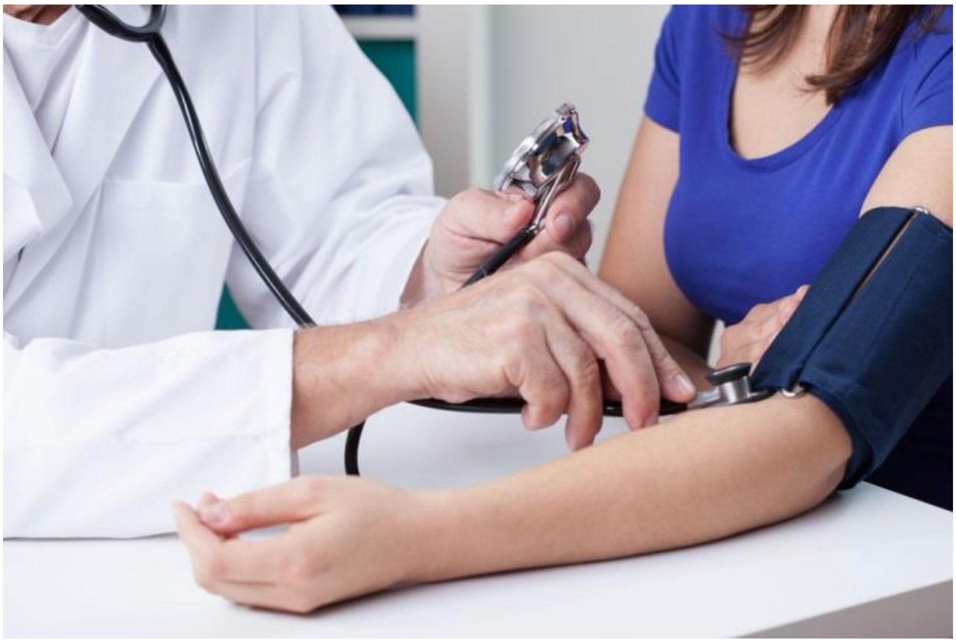

Project Managers:

Professor Dr. Volker E. Amelung
Professor Dr. Christian Krauth

Medizinische Hochschule Hannover (Hannover Medical School)
Institute of Epidemiology, Social Medicine and Health Systems Research
Carl-Neuberg-Str. 1
30625 Hannover, Germany

Cooperation Partners:

AOK Die Gesundheitskasse.
AOK Niedersachsen

KVN
Kassenärztliche Vereinigung Niedersachsen

Instructions for Completing the Questionnaire

Insert an "X" to clearly mark the box or circle indicating your response.

Boxes indicate that only ONE response can be selected. ☐ ☒ ☐

Circles mean that multiple responses can be selected. ⊗ ○ ⊗

To correct a response, completely blot out the incorrect field as follows: ■ ☐ ☒

Rectangles are completed by writing in a number: 1 5

Some questions require you to make free text responses, which should be written in block letters: Doctors

For better readability, this questionnaire was formulated using male pronoun forms only (he/him/his), but expressly refers to both males and females.

This questionnaire takes approximately 15 minutes to complete.

If you have any questions, please contact Mr. Bertolt Kuhn by phone or email: ☎ + (0)511 532 6826; ✉ Kuhn.Bertolt@mh-hannover.de.

Thanks for your assistance!

Prof. Dr. Volker E. Amelung | Prof. Dr. Christian Krauth | Bertolt Kuhn

Sources: Questions 3, 6, 7, 8, 9, 15 and 16 are based on the General Practitioner Care Survey for Municipalities in Baden-Württemberg [by KompetenzZentrum Allgemeinmedizin; Steinhäuser, Joos; Heidelberg University Hospital; 2011]. Thanks for your assistance!

A. Assessment of your county or district

Healthcare policy-makers are increasingly concerned about differences in the supply of rural areas with general practitioners and medical specialists. Some regions have a very good supply of doctors, while others report a current or upcoming shortage of physicians.

1. As a county commissioner, how satisfied or dissatisfied are you, overall, with the outpatient care available in your rural district?

Dissatisfied	Somewhat dissatisfied	Neither*	Somewhat satisfied	Satisfied
☐	☐	☐	☐	☐

2. Please rate the following statements:

	Completely disagree	Partly disagree	Partly agree	Completely agree
Overall, there are enough <u>general practitioners</u> in my county or district.	☐	☐	☐	☐
Overall, there are enough <u>specialists</u> in my county or district.	☐	☐	☐	☐
→ Which specialties are absent or under-represented?	________________			

3. Filling vacancies in medical practices is currently a problem in my county or district or will be in the near future.

Completely disagree	Partly disagree	Partly agree	Completely agree
☐	☐	☐ ↓	☐ ↓

If filling vacancies is (or might be) a problem in your county or district: **What are the reasons for this, in your opinion?** *(Multiple responses are possible.)*

- ○ General shortage of physicians
- ○ Disadvantageous geographical location of the county or district
- ○ Unattractive earning potential
- ○ High physician workload
- ○ Lack of job offers for spouses or life partners of physicians
- ○ Other (please [illegible] ________________

4. Residents in my county or district can easily reach the independent physicians practicing by public transportation.

Completely disagree	Partly disagree	Partly agree and disagree	Partly agree	Completely agree
☐	☐	☐	☐	☐

5. What are currently the greatest challenges to outpatient medical care in your county or district? *(Multiple responses are possible.)*

- ○ Reaching care-dependent patients
- ○ Accessibility of doctor's offices
- ○ Emergency care
- ○ Providing care on weekends
- ○ Filling vacancies in doctor's offices
- ○ Access to specialist physicians in the vicinity
- ○ Other (please specify): ____

B. Options for exerting municipal influence on the outpatient physician supply

In the following section, we would like to learn how you perceive generalist and specialist care as a municipal topic. What is your personal opinion?

6. How great a role does outpatient physician supply play as a location attractiveness factor for your county or district, in your opinion?

Very small	Small	Moderate	Large	Very large
☐	☐	☐	☐	☐

7. Outpatient physician supply is a fundamental public service topic in my district.

Completely disagree	Partly disagree	Partly agree and disagree	Partly agree	Completely agree
☐	☐	☐	☐	☐

8. Ensuring the outpatient physician supply is the responsibility of the Association of Statutory Health Insurance Physicians. However, county commissioners should still attend to this issue in their districts.

Completely disagree	Partly disagree	Partly agree and disagree	Partly agree	Completely agree
☐	☐	☐	☐	☐

9. What is the maximum acceptable time a resident in your district should have to travel from home to the doctor's office, in your opinion? (if not an emergency)

Please write in the maximum acceptable travel time in minutes.

To the GP:	By car	☐☐☐	Min.	By public transport	☐☐☐	Min.
To a specialist:	By car	☐☐☐	Min	By public transport	☐☐☐	Min
To the hospital:	By car	☐☐☐	Min	By public transport	☐☐☐	Min

10. Stakeholders at which level should be more involved in securing the outpatient physician supply in the future? *(Multiple responses are possible.)*

- ○ Federal / State
- ○ Municipal
- ○ Associations of Statutory Health Insurance Physicians
- ○ Health insurance companies
- ○ College and university
- ○ Other (please specify): ____________________
- ☐ No additional involvement is needed to secure the physician supply.

11. How great are your potentials to exert influence on ensuring the local outpatient physician supply as a county commissioner?

Very small	Smalll	Moderate	Large	Very large
☐	☐	☐	☐	☐

12. Do you see a need for supplementary municipal measures to encourage physicians to settle in your county or district?

☐ Yes ☐ No

13. Which supplementary municipal measures do you think are important for encouraging physicians to settle in your county or district? *(Multiple responses are possible.)*

- ○ Creating an attractive infrastructure
- ○ Easing personal framework conditions for doctors (e.g., compatibility of work and family life).
- ○ Increased cooperation with the Association of Statutory Health Insurance Physicians
- ○ Providing financial support
- ○ Other (please specify): ____________________
- ☐ No supplementary municipal measures are important / necessary for encouraging physicians to settle in my county or district?

14. Do you see any obstacles to the implementation of municipal measures to encourage physicians to settle in your county or district?

☐ Yes (please specify): *(Multiple responses are possible.)* ☐ No *Skip to Question 15*

- ◯ Lack of jurisdiction/authority or ability to intervene
- ◯ Lack of financial resources
- ◯ Lack of personnel
- ◯ Lack of knowledge
- ◯ Restrictions imposed by municipal law or competition law
- ◯ Other obstacles (please specify): ____________________

15. Please rate the <u>feasibility of implementation</u> of the following municipal measures for encouraging physicians to settle in your county or district.

	Completely unfeasible	Unfeasible	Partly feasible	Feasible	Completely feasible
Supplementary assistance for establishing a medical practice (e.g., renovation assistance)	☐	☐	☐	☐	☐
Loan acquisition support	☐	☐	☐	☐	☐
Provision of cheap properties	☐	☐	☐	☐	☐
Provision of low-rent or rent-free medical premises	☐	☐	☐	☐	☐
Creation of an information platform with lists of contacts for doctors and medical students	☐	☐	☐	☐	☐
Financial aid for medical students (e.g., scholarships)	☐	☐	☐	☐	☐
Assistance to help doctors acquire funds from support programs	☐	☐	☐	☐	☐

16. Which other municipal measures for encouraging physicians to settle in your county or district do you think are feasible?

____

____

17. Have municipal measures already been implemented to encourage physicians to settle in your county or district? If so, which measures?

18. **Would you like to have more involvement of your county or district in securing the physician supply to your area in the future?** This refers to more opportunities for cooperation or to exert municipal influence and so forth.

Strongly disagree	Disagree	Agree	Strongly agree	Undecided
☐	☐	☐	☐	☐

19. Doctors in the outpatient sector work as either independent physicians in private practice or as employees of a health care center (HCC), together with doctors of various specialties. **In the future, it should be easier for municipalities to establish health care centers as <u>municipal enterprises</u> that that employ physicians and equally participate in providing outpatient care.**
Is establishing health care centers as municipal enterprises fundamentally suitable for your county or district?

Strongly disagree	Disagree	Agree	Strongly agree	Undecided
☐	☐	☐	☐	☐

Briefly explain your response (using keywords).

C. Suitability of new health care models

Besides providing support and assistance for medical practices, various new care models are being discussed as additional ways to secure the physician supply. Some of these models are already being implemented, while others are still being treated as "visions for the future" in Germany". The degree of suitability of such care models for a given county or district varies according to the local circumstances.

20. Please rate the suitability of the following five health care models for your county or district.

I **Trained medical assistants**

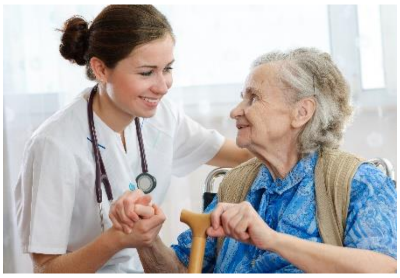

M

M

edical assistants can undergo additional training qualifying them to relieve doctors of certain duties. Routine medical duties, such as wound treatment, home check-up visits, and medical documentation, can then be performed by the Trained Medical Assistant (TMA). However, the doctor is still the responsible party. TMAs can relieve doctors of certain duties, giving physicians more time to treat patients in their office.

How suitable is this health care model for your county or district?

Very unsuitable	Unsuitable	Partly suitable and unsuitable	Suitable	Very suitable
☐	☐	☐	☐	☐

Briefly explain your response (using keywords).

2 Patient bus

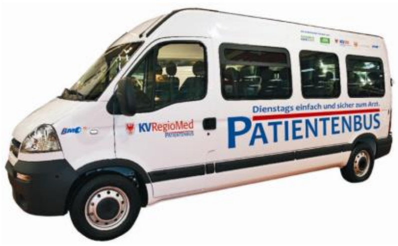

Patient buses are a means of transportation designed to ensure patient access to the doctor's offices by public transport. Patient buses take patients from remote areas of the county to the doctor’s office and back. The operating hours of the patient buses are aligned with those of the doctor’s offices.

How suitable is this health care model for your county or district?

Very unsuitable	Unsuitable	Partly suitable and unsuitable	Suitable	Very suitable
☐	☐	☐	☐	☐

Briefly explain your response (using keywords).

3 Mobile physicians’ office

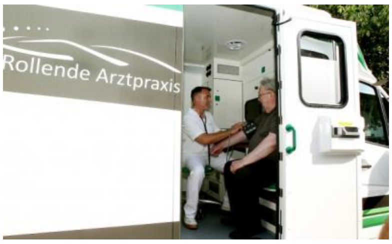

The mobile physicians’ office is a fully-equipped doctor’s office integrated in a converted camper van or minibus. The doctor can drive the mobile medical practice to remote areas where there is no local general practitioner. A mobile physicians’ office is equipped like a normal primary care practice and provides a first point of contact for medical care.

How suitable is this health care model for your county or district?

Very unsuitable	Unsuitable	Partly suitable and unsuitable	Suitable	Very suitable
☐	☐	☐	☐	☐

Briefly explain your response (using keywords).

Picture sources: KV Brandenburg (Patient bus); Fotoagentur Hübner (Mobile physicians’ office).

4 Hospital-based specialist care

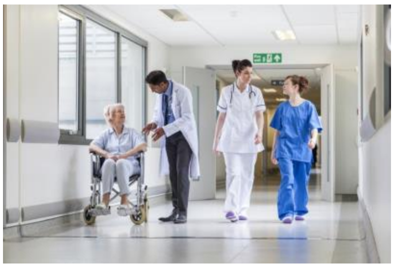

I

In regions with a scarcity of specialized physicians, local hospitals could increasingly provide outpatient services to improve access to a specialist. Patients with less serious illnesses could thus be treated in the hospital in the future. In this case, the hospital providing such outpatient care would schedule an appointment with the patient.

How suitable is this health care model for your county or district?

Very unsuitable	Unsuitable	Partly suitable and unsuitable	Suitable	Very suitable
☐	☐	☐	☐	☐

Briefly explain your response (using keywords).

5 Remote treatment (telemedicine)

I

n telemedicine, patients do not present in the doctor's office physically, but receive remote treatment and advice from a physician via telecommunication and information technology. This saves patients from having to travel long distances. Contact between telemedicine patients and doctors takes place via email, telephone and/or videoconferencing. Based on the reported symptoms of disease, the doctor decides whether to issue the patient a drug prescription or sick leave. If an examination that cannot be performed remotely is needed, the patient must schedule an appointment and present to the doctor in person.

How suitable is this health care model for your county or district?

Very unsuitable	Unsuitable	Partly suitable and unsuitable	Suitable	Very suitable
☐	☐	☐	☐	☐

Briefly explain your response (using keywords).

D. Structure of your county or district

In this last section, we ask you to provide brief information about your county or district.

21. What is the population size of your county or district?

- ☐ ≤ 80,000
- ☐ 80,001 to 120,000
- ☐ 120,001 to 160,000
- ☐ 160,001 to 200,000
- ☐ > 200,000

22. Is you county or district more urban or rural?

- ☐ Urban
- ☐ Rural

Do you have any questions or comments to the questionnaire?

____

____

____

____

____

____

____

THANKS FOR YOUR ASSISTANCE!

Please return the completed questionnaire by **15 September 2015**. Please use the postage-paid return envelope provided for that purpose.

MHH
Medizinische Hochschule
Hannover
