# Supplementary material for: Which strategies might improve local primary healthcare in Germany? An explorative study from a local government point of view
Source: BMC Fam Pract. 2017 Dec 20;18:105. doi: 10.1186/s12875-017-0696-z (PMC5738820; doi:10.1186/s12875-017-0696-z)
Supplement: Supplementary file 3 — Binary logistic regression model of the suitability of the supplement models. The regression model investigates possible connections regarding the suitability of supplement models with with indicators of a positively or negatively assessed local healthcare situation. (DOCX 15 kb) [file 12875_2017_696_MOESM3_ESM.docx]

**Supplementary File 1: Binary logistic regression model of the suitability of the supplement models**

| **Binary logistic regression model for the suitability of the supplementary models** | | | | | | | | | | | | | |
| --- | --- | --- | --- | --- | --- | --- | --- | --- | --- | --- | --- | --- | --- |
|  | | | | | |  | | | | | | | |
|  | Trained medical assistant | | | Patients’ bus | | | | Mobile physicians’ office | | | Telemedicine | | |
|  | b | SE | p-value | b | SE | | p-value | b | SE | p-value | b | SE | p-value |
| Low satisfaction with outpatient care (Ref: satisfied) | -0.749 | 0.353 | **0.034*** | -0.395 | 0.326 | | 0.226 | 0.171 | 0.379 | 0.651 | -0.506 | 0.547 | 0.355 |
| Not sufficient specialists (Ref: does not apply) | -0.163 | 0.360 | 0.651 | -0.119 | 0.321 | | 0.712 | -0.295 | 0.383 | 0.441 | -0.323 | 0.441 | 0.463 |
| Problems with replacement of physicians’ offices (Ref: does not apply) | 0.050 | 0.353 | 0.886 | 0.294 | 0.320 | | 0.359 | 0.720 | 0.411 | 0.080 | -0.191 | 0.460 | 0.678 |
| No good accessibility of physicians (Ref: good accessibility or partly / partly) | 0.660 | 0.323 | **0.041*** | -0.334 | 0.280 | | 0.234 | 0.307 | 0.329 | 0.351 | 0.072 | 0.418 | 0.864 |
| Need for municipal support in physicians settlements (Ref: no need) | 0.440 | 0.350 | 0.209 | 0.139 | 0.321 | | 0.665 | 0.061 | 0.399 | 0.878 | -0.101 | 0.457 | 0.825 |
| Sex (Ref: male) | -0.123 | 0.524 | 0.814 | 0.027 | 0.477 | | 0.954 | 0.515 | 0.505 | 0.308 | 0.615 | 0.613 | 0.315 |
| Age | 0.013 | 0.020 | 0.516 | -0.022 | 0.018 | | 0.223 | -0.035 | 0.022 | 0.108 | 0.035 | 0.028 | 0.217 |
| Spatial classification (Ref: urban or densely populated) | 0.417 | 0.369 | 0.259 | 0.910 | 0.364 | | **0.013*** | -0.081 | 0.437 | 0.852 | -0.189 | 0.491 | 0.701 |
| Constant | -0.466 | 1.233 | 0.705 | 0.478 | 1.130 | | 0.672 | 0.614 | 0.208 | 0.648 | -2.596 | 1.691 | 0.125 |
| n | 243 | | | 243 | | | | 242 | | | 242 | | |
| -2 Log-Likelihood | 281.701 | | | 324.787 | | | | 246.231 | | | 180.025 | | |
| Nagelkerke's R-Square | 0.068 | | | 0.065 | | | | 0.066 | | | 0.040 | | |
| * p < 0.05; b: regression coefficient; SE: standard error | | | | | | | | | | | | | |

**Supplementary File 1: Binary logistic regression model of the suitability of the supplement models**
